# EGCG Suppresses Adipogenesis and Promotes Browning of 3T3-L1 Cells by Inhibiting Notch1 Expression

**DOI:** 10.3390/molecules29112555

**Published:** 2024-05-29

**Authors:** Yinghao Wang, Chunfeng Li, Wenyuan Peng, Jun Sheng, Chengting Zi, Xiaoyun Wu

**Affiliations:** 1 Key Laboratory of Puer Tea Science, Ministry of Education, Yunnan Agricultural University, Kunming 650201, China; wyhh0518@163.com (Y.W.); 15154962737@163.com (C.L.); u1n3fj9u3v@163.com (W.P.); shengj@ynau.edu.cn (J.S.); 2 Department of Science, Yunnan Agricultural University, Kunming 650201, China; 3 College of Food Science and Technology, Yunnan Agricultural University, Kunming 650201, China

**Keywords:** EGCG, Notch1, UCP-1, obesity, mitochondria

## Abstract

Background: With the changes in lifestyle and diet structure, the incidence of obesity has increased year by year, and obesity is one of the inducements of many chronic metabolic diseases. Epigallocatechin gallate (EGCG), which is the most abundant component of tea polyphenols, has been used for many years to improve obesity and its complications. Though it has been reported that EGCG can improve obesity through many molecular mechanisms, EGCG may have many mechanisms yet to be explored. In this study, we explored other possible mechanisms through molecular docking and in vitro experiments. Methods: AutoDock Vina was selected for conducting the molecular docking analysis to elucidate the interaction between EGCG and Notch1, while molecular dynamics simulations were employed to validate this interaction. Then, the new regulation mechanism of EGCG on obesity was verified with in vitro experiments, including a Western blot experiment, immunofluorescence experiment, oil red O staining, and other experiments in 3T3-L1 adipocytes. Results: The molecular docking results showed that EGCG could bind to Notch1 protein through hydrogen bonding. In vitro cell experiments demonstrated that EGCG can significantly reduce the sizes of lipid droplets of 3T3-L1 adipocytes and promote UCP-1 expression by inhibiting the expression of Notch1 in 3T3-L1 adipocytes, thus promoting mitochondrial biogenesis. Conclusions: In this study, molecular docking and in vitro cell experiments were used to explore the possible mechanism of EGCG to improve obesity by inhibiting Notch1.

## 1. Introduction

Obesity is now one of the most common metabolic diseases, affecting more than 600 million people worldwide [1], and long-term obesity leads to an increased risk of diabetes [2], hyperuricemia [3], and cardiovascular disease [4]. At present, the main reason leading to obesity is excessive dietary intake and lack of physical activity, resulting in the excessive accumulation of lipids. The heat production of brown adipocytes and the browning of white adipocytes are new treatment strategies for obesity, and the browning of white fat has attracted attention in recent years due to its significant effect on fat reduction [5]. The increased expression of uncoupling protein 1 (UCP-1) mRNA and mitochondrial development in white fat are conducive to browning and can increase mitochondrial density and cell respiration [6].

Molecular docking is a computer simulation technique that simulates the interaction between a molecule and a target protein at the atomic level and calculates the affinity to evaluate the binding [7]. Molecular dynamics simulation is widely used in pharmacology and biomedical fields to study the interaction between ligands and receptors as well as conformational changes [8].

EGCG, a widely studied polyphenol, is the most abundant polyphenol in green tea [9]. It has anti-tumor [10], anti-obesity [11], anti-diabetes [12] and anti-inflammatory [13] effects. In recent years, many studies have demonstrated that EGCG can treat obesity through multiple signaling pathways. Li et al. found that EGCG can inhibit lipid accumulation in obese mice by activating AMPK [11]. Impaired bile acid (BA) synthesis leads to impaired lipid metabolism; Sheng et al. found that EGCG could alleviate obesity by regulating the bile acid signaling pathway and intestinal flora using metabolomics analysis [14]. Obesity also leads to neuroinflammation and energy metabolism disorders. Zhou et al. found that the supplementation of EGCG significantly inhibited obesity induced by a high-fat diet by enhancing brown adipose tissue (BAT) thermogenesis and reduced hypothalamic inflammation by down-regulating the hypothalamic STAT3 signaling pathway [15]. Additionally, EGCG also has significant anti-lipogenesis activity in 3T3-L1 cells [16]. Choi et al. also found that the anti-obesity effect of EGCG required the up-regulation of Beclin1-dependent autophagy and lipid catabolism in white adipose tissue (WAT) [17]. These results suggest that EGCG, as a natural polyphenol compound, has multi-target and multi-pathway characteristics in vivo due to its polyhydroxyl structure [18]. Therefore, EGCG may have many mechanisms to improve obesity yet to be explored.

Notch1, as an inflammatory marker protein, exerts regulatory effects on obesity development. Previous studies have demonstrated that the activation of Notch1 signaling promotes adipocyte proliferation [19], while the modulation of the Notch1 signal in adipose tissue macrophages is crucial for regulating inflammation and metabolism [20]. Moreover, inhibiting Notch1 expression has been shown to significantly enhance brown fat thermogenesis, promote white fat browning, and ameliorate obesity [21,22,23]. Although EGCG can improve inflammation by inhibiting Notch1 and has significant effects on obesity [11,24], it is still uncertain whether EGCG in adipose tissue can have the same metabolic-promoting and obesity-improving effects by targeting Notch1.

This study aims to investigate the potential of EGCG in inhibiting Notch1 expression, promoting adipocyte browning, and improving obesity through the establishment of a molecular docking model, conducting molecular dynamics experiments, and utilizing in vitro cell models.

## 2. Results

### 2.1. Molecular Docking

**Figure 1:**
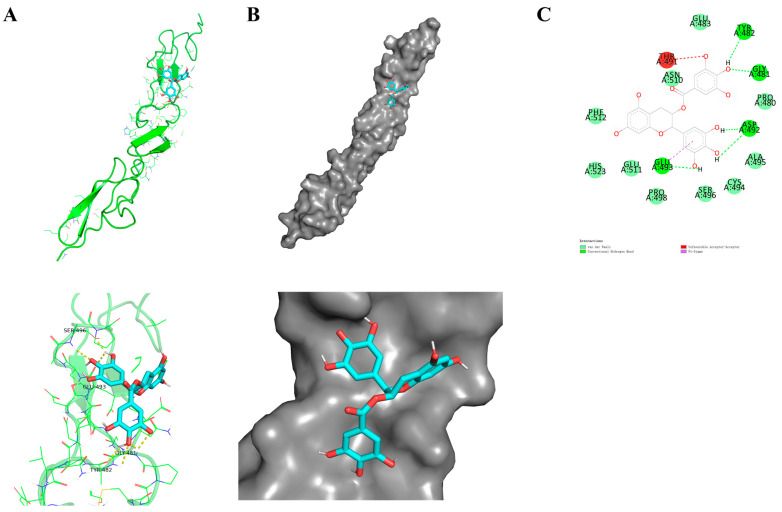
Molecular docking model of EGCG and Notch1. (**A**) The 3D model of the EGCG interaction with Notch1; (**B**) EGCG-docked Notch1 structure depicted and EGCG zoom-in binding pocket. (**C**) The 2D model of the EGCG interaction with Notch1.

Molecular docking is a theoretical method employed to investigate the interaction between receptors and ligands, enabling the prediction of the binding mode and molecular mechanism [25]. In this study, AutoDock was utilized to dock EGCG with Notch1. Degree analysis was conducted using AutoDock Vina-1.5.6 software, revealing a binding energy of −6.9 kcal/mol between Notch1 and EGCG. The binding mode of EGCG at the pocket of Notch1 is depicted in Figure 1A,B. The visual examination of the molecular docking results between EGCG and Notch1 was performed using PyMOL-1.8.6 software, demonstrating that EGCG forms hydrogen bonds with sites TYP482, GLY481, ASP492, and GLU493 on the Notch1 protein (Figure 1C). Table 1 presents the binding free energy values for the EGCG-Notch1 complex: total binding free energy (−144.317 ± 1.15 kcal/mol), van der Waals energy (−184.34 ± 1.16 kcal/mol), electrostatic energy (−32.03 ± 0.79 kcal/mol), polar solvation energy (89.13 ± 106 kcal/mol), and SASA (solvent-accessible surface area) energy (−17.06 ± 0.9 kcal/mol). These findings highlight the crucial role played by van der Waals interactions in forming the EGCG-Notch1 complex.

### 2.2. Molecular Dynamics (MD) Simulation

**Figure 2:**
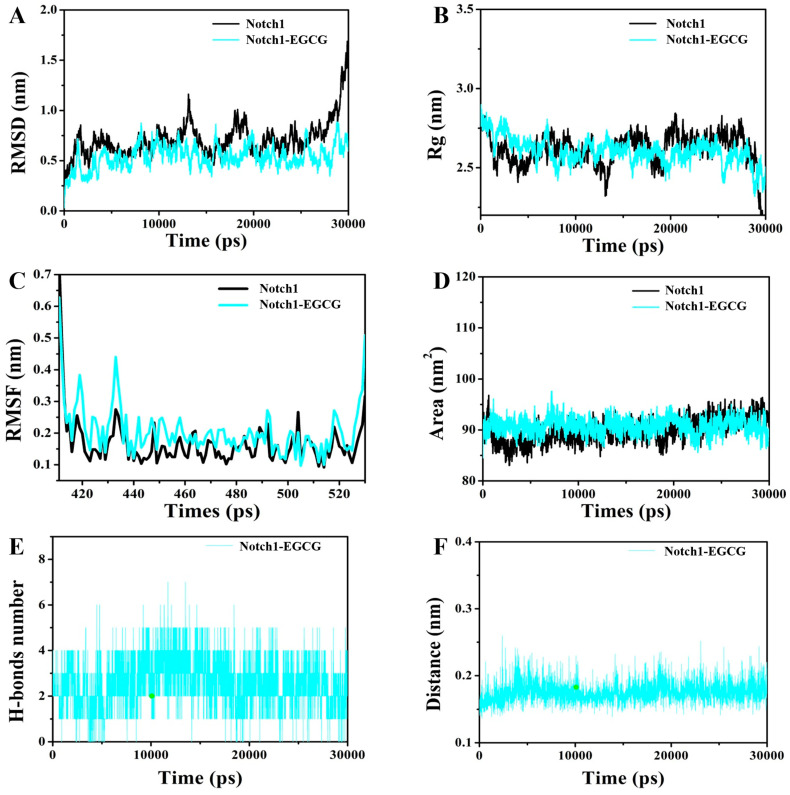
Molecular dynamics simulation of EGCG with Notch1 using the GROMACS package and the 54a7 force field at 300 K during 30 ns simulation times. (**A**) The RMSD of carbon alpha plot for Notch1 and the EGCG-Notch1 complex. (**B**) The Rg plot for Notch1 and the EGCG-Notch1 complex. (**C**) The RMSF plot for Notch1 and the EGCG-Notch1 complex. (**D**) The SASA plot for Notch1 and the EGCG-Notch1 complex. (**E**) The number of hydrogen bonds formed between EGCG and Notch1 during the simulation. (**F**) The distance of hydrogen bond formed between EGCG and Notch1.

The stability of the ligand-protein system was analyzed at different time scales using MD simulations to further investigate the interaction between Notch1 and EGCG. Molecular dynamics simulations were performed for 30 ns using the GROMAC 5.1.2 software package [26]. Figure 2A demonstrates that the α-C RMSD values of free Notch1 are comparable to those of the Notch1-EGCG complex. However, after 25 ns, there is a larger fluctuation in α-C RMSD values for Notch1, while the α-C RMSD values for the Notch1-EGCG complex exhibit smaller fluctuations, indicating enhanced stability upon the binding of EGCG to Notch1. The Rg value reflects the compactness of a complex (Figure 2B), where compared to Notch1 alone, a decrease in Rg is observed after 20 ns for Noch1-EGCG, suggesting tighter structure formation in the EGCG-Notch1 complex. The trajectory-based RMSF plot of the EGCG-Notch1 complex was utilized to analyze results from 20 ns to 30 ns (Figure 2C), with exceptional stability among interacting residues indicated by an RMSF value of 0.15 Å. The SASA value of the complex indicates the solvent polarizability energy of molecular interactions, as illustrated in Figure 2D. It is significantly higher for Notch1 binding with EGCG compared to Notch1 alone, suggesting that upon binding with EGCG, the structure of Notch1 undergoes contraction and forms a more stable complex. As depicted in Figure 2E, hydrogen bonding between EGCG and Notch1 occurs from the initial stages and gradually intensifies within the range of 0~7. Furthermore, as shown in Figure 2F, the calculated distance between EGCG and Notch1 reaches a minimum value of 0.16 nm. These results further validate the findings from docking studies, emphasizing the crucial role played by hydrogen bonding in mediating interactions between the EGCG-Notch1 complex.

### 2.3. EGCG Structural Formula, EGCG Cytotoxicity to 3T3-L1 Cells

**Figure 3:**
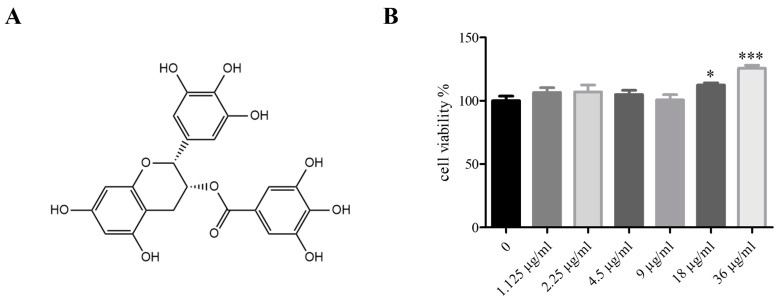
The structural formula of EGCG and the cytotoxicity of EGCG to 3T3-L1 cells. (**A**) Structural formula of EGCG. (**B**) MTT showed the cytotoxicity of EGCG at different concentrations in 3T3-L1 cells. The values are expressed as means ± SEM (n = 6). * *p* < 0.05 and *** *p* < 0.001 compared with the 0 μM EGCG.

Before starting the experiment, we investigated the cytotoxicity of EGCG to 3T3-L1 cells through cytotoxicity tests, and it was found that EGCG concentrations (2.5–80 μM) did not produce cytotoxicity in 3T3-L1 cells (Figure 3B).

### 2.4. Effect of EGCG on Lipid Synthesis during 3T3-L1 Cell Differentiation

**Figure 4:**
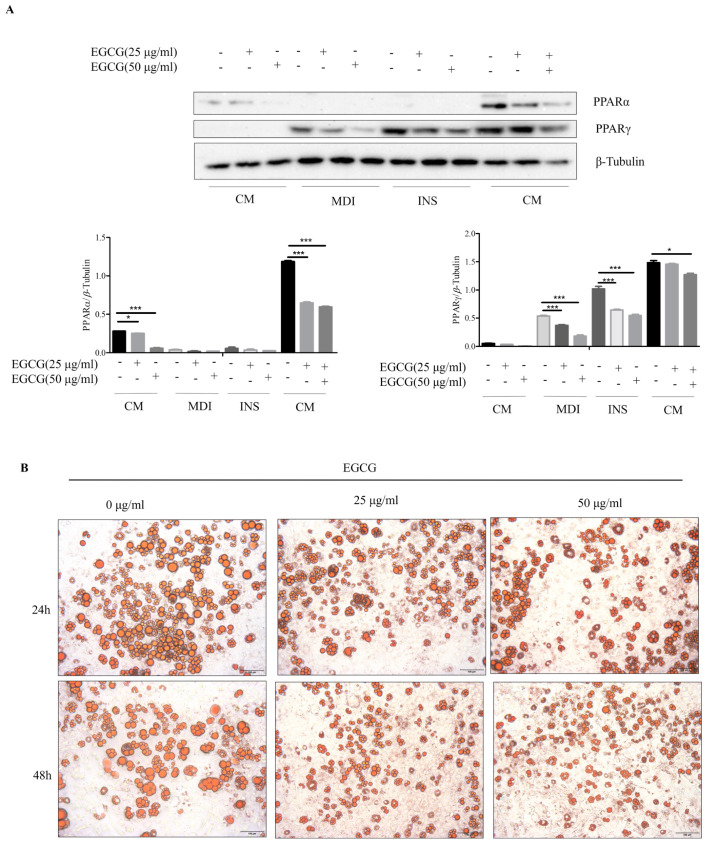
Effect of EGCG on lipid synthesis during 3T3-L1 cell differentiation. (**A**) Western blot analysis showed the expression levels of PPARγ and PPARα protein in 3T3-L1 cells treated with different concentrations of EGCG at different differentiation stages. (**B**) Oil red O staining in 3T3-L1 cells treated with different concentrations of EGCG at 24 h and 48 h. The values are expressed as means ± SEM (n = 3). * *p* < 0.05 and *** *p* < 0.001 compared with the other group.

PPARγ and PPARα are key proteins in fatty acid synthesis [27], and the expressions of PPARγ and PPARα gradually increased with the differentiation stages of 3T3-L1 cells. After EGCG treatment, the expressions of PPARγ and PPARα at each stage were significantly inhibited in a dose-dependent manner (Figure 4A). After the treatment of 3T3-L1 cells with different doses of EGCG, the lipid droplet sizes of 3T3-L1 cells were significantly reduced in a dose- and time-dependent manner (Figure 4B). These results suggested that EGCG treatment significantly inhibited lipid synthesis during 3T3-L1 cell differentiation and reduced lipid droplet sizes.

### 2.5. Effects of EGCG Treatment on Notch1 and UCP1 Expression in 3T3-L1 Cells

**Figure 5:**
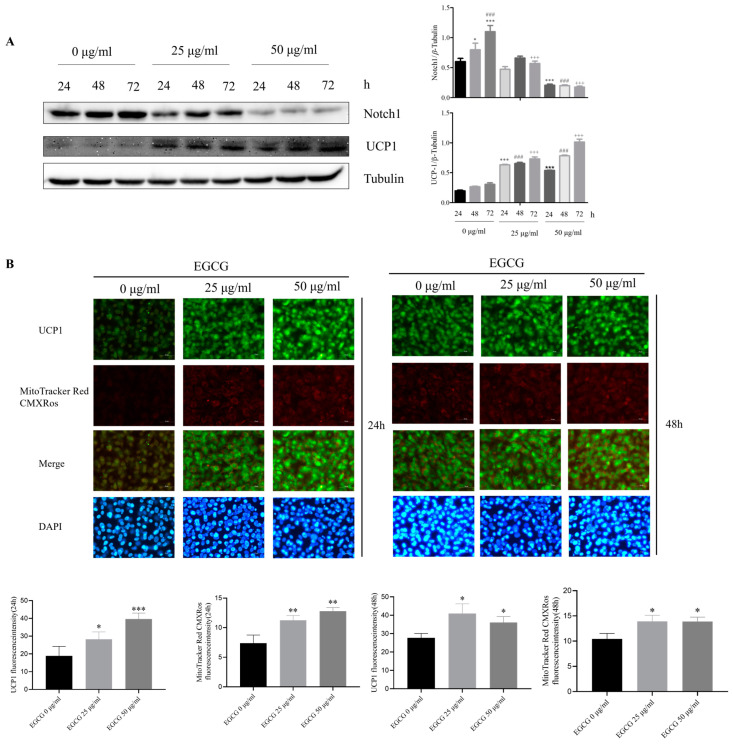
Effects of EGCG treatment on Notch1 and UCP-1 expression in 3T3-L1 cells. (**A**) Western blot analysis showed Notch1 and UCP-1 protein levels in 3T3-L1 cells treated with different concentrations of EGCG for 24 h. (**B**) UCP-1 immunofluorescence staining (green). Mitochondrial fluorescence staining (red) micrographs showed UCP-1 expression and mitochondrial biogenesis in 3T3-L1 cells treated with different concentrations of EGCG for 24 h. The values are expressed as means ± SEM (n = 3). * *p* < 0.05, ** *p* < 0.01 and *** *p* < 0.001 compared with the 0 μg/mL-24 h group, ### *p* < 0.001 compared with the 0 μg/mL-48 h group, and +++ *p* < 0.001 compared with the 0 μg/mL-72 h group.

UCP-1 is one of the marker proteins of mitochondrial genesis [28]. Previous studies have shown that the increased expression of UCP-1 can significantly inhibit fat synthesis, and the expression of Notch1 is also related to UCP-1 [21]. After the EGCG treatment of mature 3T3-L1, the expression level of Notch1 decreased significantly with the increasing treatment time; in contrast, UCP-1 increased significantly with the increasing EGCG treatment time (Figure 5A). UCP-1 immunofluorescence and mitochondrial staining also showed the same results; EGCG significantly enhanced the expression of UCP-1 in 3T3-L1 cells and promoted mitochondrial genesis (Figure 5B). These results suggested that EGCG could promote UCP-1 expression and mitochondrial genesis by inhibiting Notch1 expression.

## 3. Discussion

With the improvement in living standards and the changes in lifestyles, the number of obese people has increased year by year, and obesity is accompanied by various complications, which has attracted more and more attention around the world. Obesity is also a major factor in many chronic metabolic diseases, including atherosclerosis [29], non-alcoholic fatty liver disease [30], and chronic kidney disease [31]; therefore, improving obesity can effectively prevent a variety of chronic metabolic diseases. Meanwhile, studies have shown that inhibiting GATA3 in adipocytes can significantly inhibit adipose differentiation and improve insulin resistance and T2DM induced by obesity [32]. At present, the treatment for obesity is mainly dependent on changes in lifestyles and energy intake reduction, with drug intervention usually used in severe cases [33]. In recent years, with the popularity of the concept of food and medicine homology, people have paid more and more attention to seeking effective ingredients from food to prevent chronic diseases [34]. Many studies have shown that many foods with the same origin as food and medicine can improve obesity. Wang et al. found that puerarin in pueraria can prevent obesity caused by a high-fat diet by regulating intestinal flora [35]. *Moringa oleifera*, which is a homologous food and medicine, has also been shown to alleviate obesity caused by a high-fat diet by regulating intestinal flora [36]. Tea, as one of the most commonly consumed drinks in the world, contains many potent ingredients that can improve obesity [37,38]. As the tea variety containing the most tea polyphenols, green tea has been proven to have a significant improvement effect on obesity by many studies [39,40] and is related to a variety of effective components contained in it, including EGCG, ECG, EC, etc. [41], and there may be synergistic effects. In addition, while green tea extract produces effects, various polyphenols contained in it may be mutually complementary or have the same effect. Chendi Zhan et al. found that EGCG and EGC both have the effect of improving Alzheimer’s disease [42], while EGCG and ECG can significantly inhibit melanoma proliferation [43]. However, as the most abundant and effective of tea polyphenols, EGCG [44] plays a key role in the improvement of obesity and other metabolic diseases by green tea extract [45,46]. As the most abundant component of tea polyphenols, EGCG has been shown to improve many diseases, including cancer, arthritis, diabetes, and colitis [47,48,49,50,51]. Many studies have proven that EGCG has an improving effect on obesity [11,52]. The purpose of this study is to explore some possible mechanisms of EGCG on obesity through the combination of molecular docking, molecular dynamics, and in vitro experiments. The results showed that EGCG could inhibit adipocyte fat synthesis, promote mitochondrial biogenesis, and promote white fat browning by inhibiting Notch1 expression.

Studies have shown that fat cell hypertrophy promotes cell rupture through physical reasons, thereby causing inflammation [53] and that the ectopic accumulation of lipids, mainly in the liver, is the main cause of insulin resistance and inflammation [54,55]. A previous study also showed that the occurrence of inflammation not only reduced the action of insulin but also decreased the heat production of brown adipocytes and beige adipocytes [56]. The enhanced thermogenic activity of brown fat and the browning of white adipocytes can prevent obesity and related metabolic disorders. However, excessive obesity would aggravate local inflammation of white adipocytes and brown adipocytes, impair energy consumption and glucose absorption, and, thus, directly change the thermogenic activity of brown adipocytes and the browning process of white adipocytes [57]. Notch1 is a key target of inflammation [58], and is associated with many inflammatory pathways. Studies have shown that inhibition of the Notch1-PI3K/AKT signaling pathway can inhibit inflammation and immune response induced by nitric oxide [59]. Notch1 has also been extensively studied as a key receptor for EGCG in many diseases [60,61,62]. These results and studies further suggest that the improvement of EGCG on obesity is strongly related to Notch1. To further confirm our hypothesis, we used molecular docking to demonstrate that EGCG is hydrogen bonded to the Notch1 protein. The results of molecular dynamics indicate that the complex structure formed by EGCG and Notch1 is more compact compared to Notch1. EGCG has been proven to have an improving effect on obesity. We first used the oil red O experiment for verification, and the results showed that EGCG treatment for 24 h and 48 h could significantly reduce the sizes of lipid droplets in 3T3-L1 adipocytes in a dose-dependent manner. EGCG also significantly improved lipid accumulation during 3T3-L1 cell differentiation. Our results showed that EGCG treatment could significantly inhibit the expression of PPARγ and PPARα proteins during the differentiation of 3T3-L1 cells. These results indicate that EGCG has a significant ameliorative effect on obesity.

**Figure 6:**
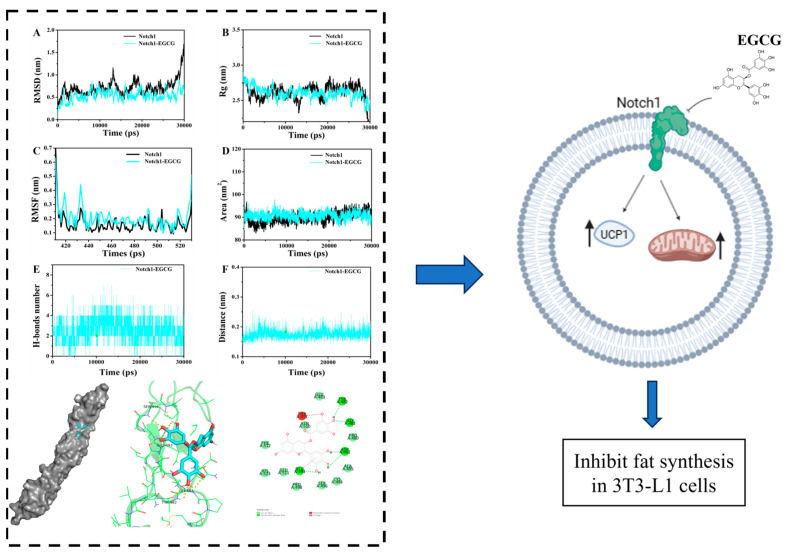
In summary, combined with molecular dynamics simulation and molecular simulation docking, EGCG promotes mitochondrial biogenesis and UCP-1 expression in 3T3-L1 adipocytes by inhibiting Notch1 expression, thereby promoting white adipocytes browning and improving obesity. Image created with Biobender.com.

UCP-1, as a marker protein for mitochondria, is also a marker protein for the browning of white fat [28]. We have previously discussed the effects of inflammation on brown fat thermogenesis and the browning of white adipocytes and discussed the role of Notch1 in this process. Due to the close relationship between EGCG and Notch1, we demonstrated in vitro experiments that EGCG treatment can significantly inhibit the expression of Notch1 in 3T3-L1 adipocytes while enhancing the expression of UCP-1. Mitochondrial staining can clearly express mitochondrial biogenesis and is also one of the signs of the browning of white adipocytes [63]. Our results show that EGCG treatment can significantly enhance mitochondrial genesis and UCP-1 expression in 3T3-L1 adipocytes. These results show that EGCG can enhance UCP-1 expression and mitochondrial biogenesis by inhibiting Notch1 expression, thereby promoting the browning of white adipocytes to achieve the purpose of ameliorating obesity (Figure 6). This study used molecular simulation docking and in vitro experiments to verify that EGCG inhibits the expression of Notch1 by binding to Notch1, thus promoting the browning of adipocytes. However, as a polyphenol, EGCG has been proven to have a variety of biological activities and biological targets. It has been found that EGCG can improve obesity in various ways, including reducing inflammation levels [15] and activating the AMPK signaling pathway [11], and it has been proven that EGCG can prevent obesity by up-regulating adipocyte autophagy [64]. Therefore, EGCG has many effects on improving obesity and promoting the browning of white fat.

## 4. Materials and Methods

### 4.1. Molecular Docking

The predicted target proteins were molecular-docked with EGCG. The proteins were downloaded from the PDB database (http://www.rcsb.org). The water molecules and ligands were removed using PyMOL, and then the target proteins were imported into AutoDockTools 1.5.6 for hydroprocessing. EGCG was imported as a ligand and then simulated docking with the target proteins and stored in PDBQT format [65]. The results were visualized with PyMOL and Discovery Studio 2019 Client.

### 4.2. Molecular Dynamics (MD) Simulation

The MD simulations of Notch1 and EGCG-Notch1 complex were conducted using the GROMACS 5.1.2 software package, employing the GROMACS 54a7 force field [66,67]. The solvation of Notch1 and EGCG-Notch1 complex systems was performed with a simple point charge (SPC) water model, followed by their placement within a cubic box filled with water molecules [68]. To maintain a distance of 1 nm from the edge of the box, counterions (Na^+^) were added to neutralize the surface charges of both complexes. Subsequently, energy minimization was carried out for 50,000 steps to optimize the prepared systems. Equilibration was achieved through normal volume and temperature (NVT) ensemble at 300 K for 100 ps, followed by density equilibration under normal pressure and temperature (NPT) conditions at 300 K over another 100 ps duration. Finally, an MD simulation was performed for a total time span of 30 ns with a time step size set as 2.0 fs. Various structural properties including root mean square deviation (RMSD), root mean square fluctuation (RMSF), radius of gyration (Rg), solvent accessible surface area (SASA), hydrogen bond formation between ligand and protein along with its distance measurement, and principal component analysis (PCA) were calculated to analyze system dynamics. Additionally, the secondary structure analysis of the EGCG-Notch1 complex employed a defined secondary structure assignment algorithm provided by the do_dssp tool.

### 4.3. Cell Culture and Differentiation

The 3T3-L1 murine preadipocytes were purchased from Kunming Cell Bank, Kunming Institute of Zoology, Chinese Academy of Sciences. The 3T3-L1 preadipocytes were cultured using Dulbecco’s Modified Eagle Medium (DMEM) and supplemented with 10% fetal bovine serum (FBS, Gibco) containing penicillin G sodium salt 100 U/mL. Streptomycin sulfate, in a 0.1 mg/mL volume, (Meilunbio, Dalian, China) was cultured in a 5% CO_2_ incubator at 37 °C. After 5 days of culture, 3T3-L1 cells were treated with 10% FBS and 0.5 mmol/L 3-isobutyl-1-methylxanthine (IBMX, Yuanye, Shanghai, China), 2.5 mmol/L Dexamethasone (Dex, Sigma-Aldrich, St. Louis, MI, USA), and 8.7 mmol/L insulin were cultured in DMEM (MDI) medium for 2 days. Then, the DMEM (INS) medium containing 10% FBS and 10 mmol/L insulin was replaced and cultured for 2 days. Subsequently, DMEM (CM) medium containing 10% FBS was replaced every 2 days until 3T3-L1 cells differentiated and matured.

### 4.4. Western Blot Analysis

Cell proteins were extracted using RIPA buffer (Solarbio, Beijing, China). The protein concentration was detected using the BCA method. Proteins were isolated using 10% SDS-PAGE electrophoresis. It was then transferred to a polyvinylidene fluoride (PVDF) membrane (Merk Millipore, Burlington, MA, USA), sealed with 5% skim milk powder at room temperature for 1 h, followed by primary antibody at 4 °C overnight. It was washed with TBST solution 3 times, 5 min each time, and incubated with 2 antioxidants containing horseradish peroxidase at room temperature for 1 h. Finally, the protein bands were detected using supersensitive enhanced chemiluminescence substrates and quantified using ImageJ 1.46v.

### 4.5. Oil Red O Staining

After the drug treatment, the cells were washed twice with PBS, fixed at room temperature with 4% paraformaldehyde for 20 min, washed twice with PBS, and stained with Oil Red O solution for 15 min. The photomicrograph was obtained through a microscope (OLYMPUS CKX41, Shanghai, China).

### 4.6. Cellular Immunofluorescence

The 3T3-L1 adipocytes were treated with EGCG and then subjected to immunofluorescence assay. After fixation with 4% paraformaldehyde and blocking with 5% skim milk powder, the primary antibody was incubated at 4 °C overnight, followed by a FITC-coupled secondary antibody incubated at room temperature for 1 h Finally, after being fixed at room temperature with a DAPI sealer. Images were observed and obtained using a fluorescence microscope (Leica DM2000, Solms, Germany).

### 4.7. Statistical Analysis

Data are presented as the means ± standard error of the mean (SEM). One-way analysis of variance (ANOVA) and *t*-tests were performed using the SPSS 13.0 software package. A value of *p* < 0.05 was considered to be statistically significant.

## 5. Conclusions

According to the key role of inflammation in the development of obesity, EGCG has a resistant activity. And, we found that EGCG could bind to Notch1 through molecular docking and molecular dynamics experiments. Moreover, in vitro experiments, it was proved that EGCG could promote the mitochondrial biogenesis and UCP-1 expression of adipocytes by inhibiting Notch1 expression, so as to promote the browning of white adipocytes and improve obesity.

## Figures and Tables

**Table 1:** Calculated MM-PBSA binding free energy in kcal/mol for the Notch1-EGCG complex.

Energy Contribution	Value (kcal/mol)
ΔG_vdw_ ^a^	−184.34 ± 1.16
ΔG_Electr_ ^b^	−32.03 ± 0.79
ΔG_Polar_ ^c^	89.13 ± 1.06
ΔG_SASA_ ^d^	−17.06 ± 0.90
ΔG_bind_ ^e^	−144.30 ± 1.15

^a^ ΔG_vdw_ is van der Waals energy; ^b^ ΔG_Electr_ is electrostatic energy contribution; ^c^ ΔG_Polar_ is polar contributions between the solute and solvent to the solvation energy; ^d^ ΔG_SASA_ is non-polar solvation energy using the solvent accessible surface area; ^e^ and ΔG_bind_ is the total free binding energy.

## Data Availability

All data used in this study are included in this article.
